# Investigating the level of education in the field of occupational safety and health in preparation for future profession: a Slovak case study

**DOI:** 10.3389/fpubh.2026.1744127

**Published:** 2026-04-23

**Authors:** Peter Brečka, Ivana Tureková, Michal Munk

**Affiliations:** 1 Department of Technology and Information Technologies, Faculty of Education, Constantine the Philosopher University, Nitra, Slovakia; 2 Department of Informatics, Faculty of Natural Sciences and Informatics, Constantine the Philosopher University, Nitra, Slovakia; 3 Science and Research Centre, University of Pardubice, Pardubice, Czechia

**Keywords:** pupils, questionnaire survey, risk management, teachers, training in occupational safety

## Abstract

**Introduction:**

This article presents a critical overview of education in the field of occupational safety and health (OSH) among pupils, along with the perspectives of secondary school teachers in the Slovak Republic. It outlines the contextual background, identifies key challenges, and defines the scientific approach to the issue, with particular attention to the specific conditions of OSH education in Slovakia.

**Methods:**

To evaluate the quality of OSH training within the dual education system, a questionnaire survey was conducted among teachers and pupils. A stratified sampling method was applied, and responses were collected anonymously. The sample consisted of 374 pupils (35% female, 65% male), aged 15–19, and 90 teachers (40% female, 60% male).

**Results:**

The findings revealed several significant issues. Pupils reported insufficient explanations of alcohol testing procedures, with differences observed across study programmes (ranked: hotel services, business services, technical and information technology services). Both teachers and pupils expressed generally neutral attitudes toward engagement in training. However, multiple comparisons across items and factor combinations indicated a lower level of proficiency in risk analysis training. Statistically significant differences were also identified in relation to position, gender, experience, and study programme.

**Discussion:**

The results highlight gaps in the effectiveness of OSH training within the Slovak dual education system, particularly in risk analysis competencies and clarity of procedural instruction. These findings suggest the need for targeted improvements in training content and delivery, taking into account demographic and educational differences among participants.

## 1 Introduction

According to the European statistics on occupational accidents (1, 2) young people aged up to 24 years have a higher probability of experiencing a work-related accident resulting in more than 3 days of incapacity for work (3, 4). The standardized rate of accidents among young individuals is 1.3 to 1.4 times higher compared to older employees. One of the reasons for the higher risk is that new starters have less experience and are less trained (5, 6).

Just like in other European countries, employers in the Slovak Republic are required to provide their employees with occupational safety and health training (7). The specific content and extent of this training are regulated by law (8).

Apprentices participating in dual education training programmes must complete OSH training (9–12). Similarly to employees, they must be knowledgeable about OSH regulations and safety guidelines for their specific tasks (13–16). This training encompasses theoretical knowledge, understanding of their rights and duties, preventive measures, safety procedures for operating machinery and equipment, safe workplace conduct, work-related injuries, prohibited actions, and emergency response procedures (17–20). Hygiene standards and assessments of the workplace environment must also be included in the training (21). Practical training enables apprentices to acquire and develop the skills needed for their future profession (22, 23). OSH training is designed to equip pupils with the knowledge and skills necessary to identify hazards, change their attitudes, and develop safe work habits, thereby preventing work-related accidents (24, 25). The safety habits learned during vocational training continue to be applied throughout their careers (26).

Despite the recognised importance of occupational safety and health education for young workers, limited research has examined how OSH training is perceived by pupils in dual vocational education systems and by the teachers responsible for their preparation. Understanding these perceptions is important for identifying potential weaknesses in the content and delivery of safety training and for improving the preparation of pupils for safe professional practice.

This study examines the perceptions of pupils and teachers regarding the quality and content of occupational safety and health (OSH) training in formal education within the Slovak dual education system. Data were analysed by target group, gender, field of study, and teaching experience. The study identified strengths and weaknesses of the programme to inform its improvement. To address the aim of the study, the following research questions were formulated:

*QR1*: Are there differences in attitudes towards OSH education among teachers/pupils, between fields of study, genders of respondents, and experience/length of practice?

*QR2*: Is information about the most significant hazards and risk assessments included in the training?

*QR3*: Do the trainings meet the didactic principles of clarity, the connection between theory and practice, and feedback?

The questionnaire survey was designed to assess the level of knowledge among respondents, which can have a significant impact on the performance of safe work. We also explored whether the training was interesting, engaging, and included specific examples of good practice.

The contributions of this paper are as follows:

(a) Identifying the areas of education perceived as the “weakest” by the research group of respondents, and proposing measures to improve the methods and approaches to education in these areas.
(b) Identifying differences in perceptions of the education between pupils and teachers, and between genders. Subsequently, applying an individualized approach to different age and gender groups in further education, selecting appropriate teaching methods, duration of education, etc.
(c) Analysing education systems to identify and assess occupational hazards and risks.
(d) Improving the quality of education for target groups by using active learning methods.
(e) Pupils in dual education are shaping their attitudes towards safety for their future professional lives. The content and approaches to training can significantly influence the transfer of learning to the workplace (27, 28).

To ensure that occupational safety and health training has a positive impact on workers (e.g., increasing knowledge, changing attitudes, improving behaviour, or protecting health), it is essential to use innovative educational methods and continuously improve the quality of training (29, 30).

## 2 Materials and methods

The study adopted a quantitative research methodology, starting with rigorous data collection procedures followed by data analysis.

The aim of the research was to investigate, through a questionnaire survey, whether the methods, content and forms of OSH education in secondary schools participating in the dual education system are at a sufficient level and to what extent pupils are able to apply theoretical knowledge in practice. Questions on the knowledge of specific job-related risks were essential. The questionnaire survey also included the opinions of teachers. We were interested in the perspective of educators, how they perceive the quality of occupational safety training and its effectiveness in ensuring the safety of pupils in the workplace. The validation of the questionnaire was carried out through pilot testing involving students and teachers from selected vocational schools in each region of Slovakia. Subsequently, necessary revisions were incorporated.

### 2.1 Data collection

In order to obtain a more representative spectrum of respondents, we used stratified sampling and the option of anonymous questionnaire completion through Google Forms. At the end of 2022, communication was initiated with the headmasters of 111 secondary vocational schools in the Slovak Republic, requesting the distribution of a link to the online survey. The survey data collection process took 3 months (January 2023 to April 2023).

### 2.2 Questionnaire survey

The questions designed for teachers and pupils can be found in Appendix A. In addition to collecting demographic information, the research questionnaires for pupils and teachers also included a total of 17 closed-ended questions primarily focused on evaluating the methods, forms, and content of OSH education. The questionnaires also included questions related to identifying the most significant workplace hazards, procedures in case of accidents, and suspected alcohol use. At the end of the questionnaires, there was space for respondents (both teachers and pupils) to express their opinions and provide recommendations to improve the quality of OSH training.

### 2.3 Research sample

The characteristics of the study population are summarized below. A total of 374 pupils (35% female and 65% male) and 90 teachers (40% female and 60% male) participated in the questionnaire survey. The responses were evaluated based on multiple criteria. Firstly, from the perspective of the pupils, and secondly from the perspective of the teachers. Other examined factors included gender, length of teaching experience, pupil age, and study programmes taught or studied. Due to a wide range of different study disciplines, they were categorized into the fields of technical and information technology services, business services, hotel services, and other services. Furthermore, the responses were analysed based on the grade levels in which the teachers teach and the pupils study.

### 2.4 Evaluation of answers

A structured research questionnaire was designed to measure the opinions of the respondents. The variables were scaled using a five-point Likert scale ranging from “1 = strongly disagree” to “5 = strongly agree.” The Likert scale was designed to allow respondents to select the option that best aligns with their opinion (31).

The internal consistency of the questionnaire was assessed using Cronbach’s alpha coefficient. The calculated value (*α* = 0.884) indicates good internal reliability of the measurement instrument. The questionnaire consisted of 17 Likert-scale items evaluating different aspects of occupational safety and health training.

Although Likert-scale responses represent ordinal data, the use of parametric statistical tests is commonly accepted in social science research when the scale contains five or more response categories and the sample size is sufficiently large. In this study, the assumption of normality was assessed using normal probability plots. Given the relatively large sample size (374 pupils and 90 teachers), parametric statistical procedures were considered appropriate for the analysis. Previous methodological studies have also shown that parametric tests are robust to moderate deviations from normality when applied to Likert-scale data with large samples (32).

## 3 Results

This section presents the key findings related to the research questions. Descriptive statistics and hypothesis testing results are presented in the following subsections, while detailed response profiles for specific combinations of factors are provided in the appendices.

**Figure 1:**
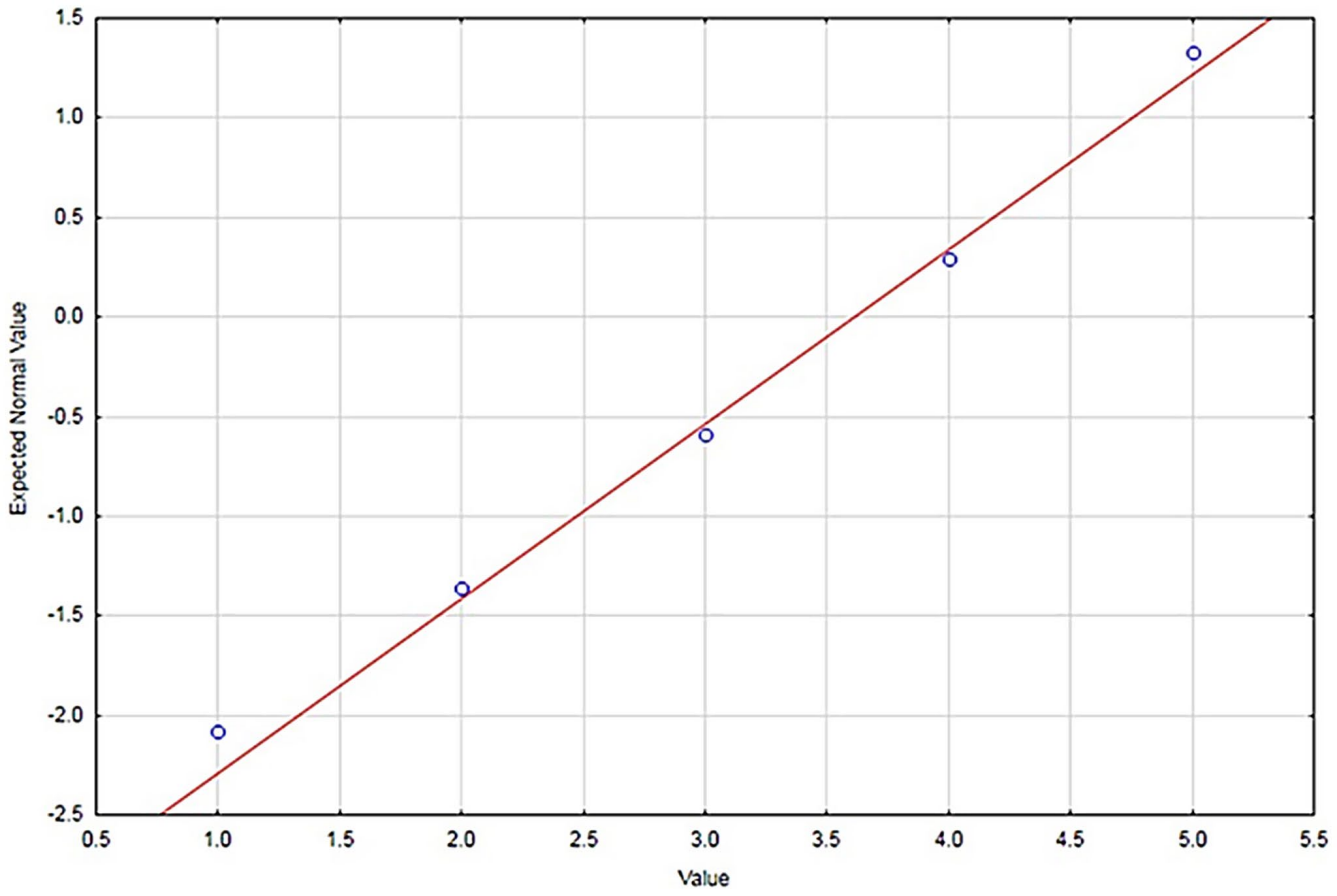
Normal probability plot for the item “Training - risk analysis”.

To verify the assumption of normality, normal probability plots were used, which visualize the observed values of the investigated variables and the expected normal values. Normality was also examined for individual levels of factors and their combinations, such as theoretical knowledge for the teacher–male group. Presenting all combinations would result in a large number of graphs; therefore, only representative plots for the examined variables are presented. The assumption of normality was confirmed for the investigated variables, including the levels of factors and their combinations, with only minor deviations observed (e.g., Figure 1).

### 3.1 Descriptive statistics

The results from the tables of descriptive statistics indicate potential differences within the individual examined items as perceived by teachers and pupils. The graphs visualize the point and interval estimates of the mean.

**Figure 2:**
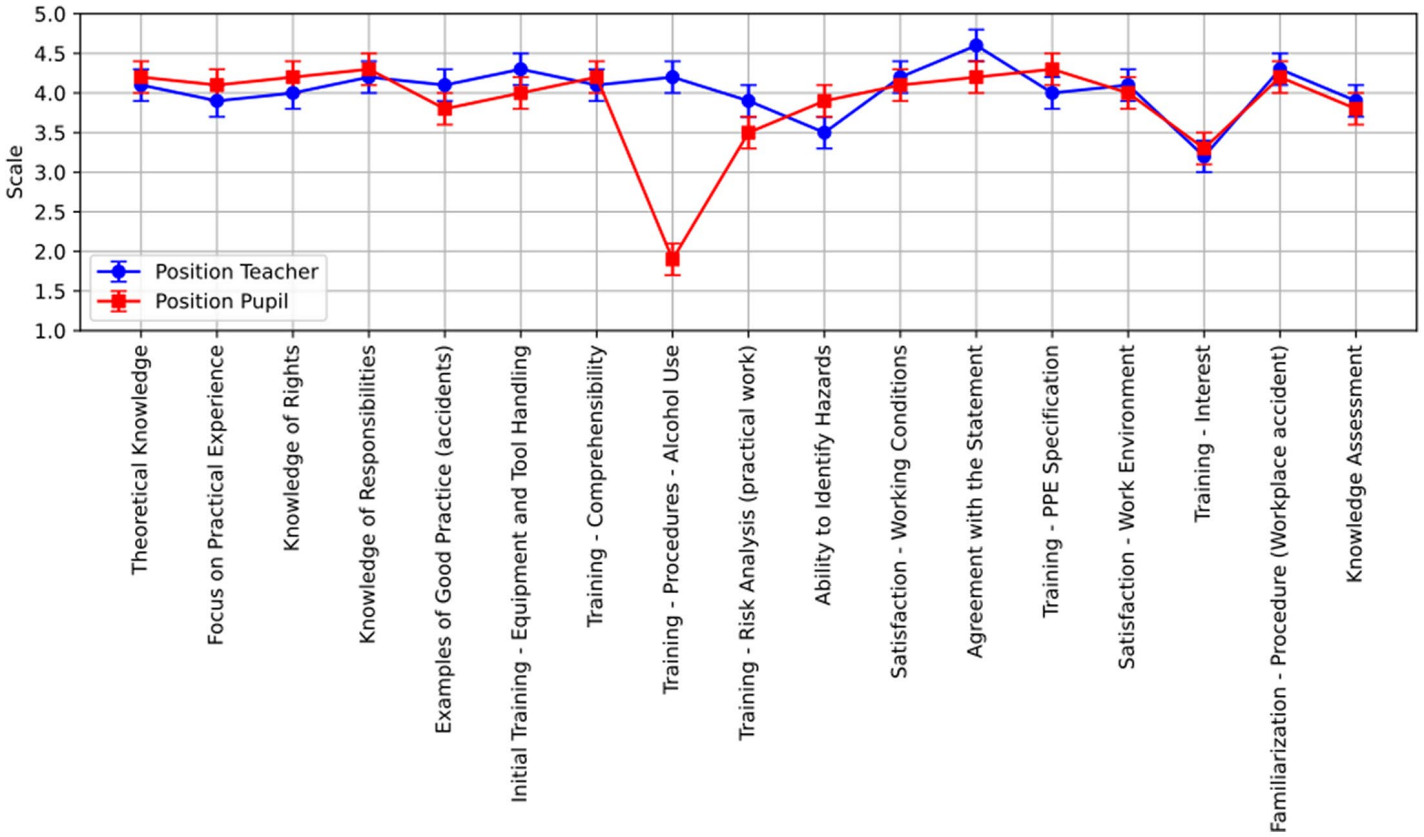
Responses of teachers and pupils to individual items.

In Figure 2, we can observe more significant disparities in responses, particularly within the category of position - the perspective of pupils. This relates to the item that examines whether pupils are informed about the process of identifying and the consequences of alcohol use as part of their training. Teachers assess this topic as adequately explained, whereas pupils disagree with this statement.

**Figure 3:**
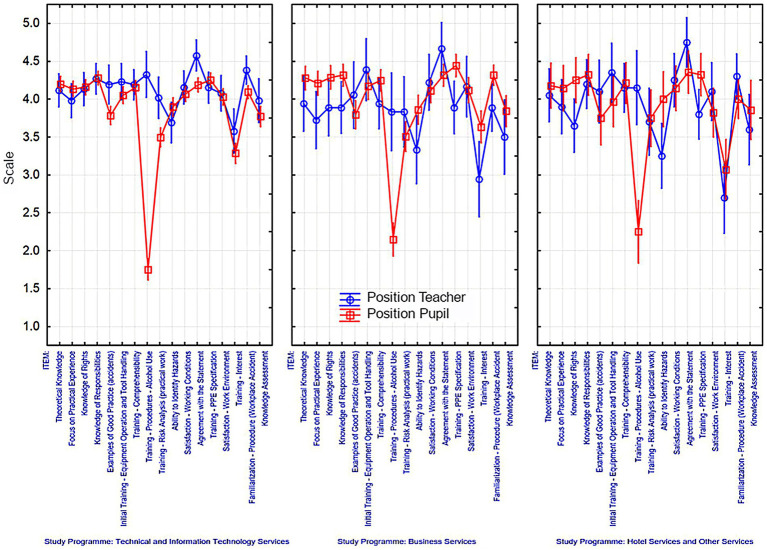
Responses to individual items from the perspective of study programmes.

When making an evaluation from the perspective of study programmes (as shown in Figure 3), where the research was conducted, similar patterns were observed to those of the previously mentioned factors. The majority of responses were found in the range of 3.5 to 4, indicating agreement or complete satisfaction with the provided quality of education in the field of OSH from the perspective of both teachers and pupils. This also implies the ability to apply the acquired theoretical knowledge in practice.

Minor differences can be seen in partial dissatisfaction when it comes to the perceived interest in the trainings, particularly in the field of hotel and other services, as well as in the field of business services from the perspective of teachers. Similarly to the previously mentioned figures, there is a prevailing dissatisfaction from the pupils’ side regarding the trainings focused on procedures related to alcohol use, especially in the field of technical and information technology services.

Teachers in the field of technical and information technology services expressed the highest satisfaction with the familiarization of procedures related to occupational accidents. On the other hand, in business, hotel, and other services study programmes teachers rate the initial trainings on equipment operation and tool handling the most positively. Teachers also expressed satisfaction with working conditions and workspaces.

### 3.2 Hypotheses

In addition to descriptive statistics, inferential statistical analyses were applied to examine differences between respondent groups and combinations of factors. The analysis further included Mauchly’s test of sphericity, the Greenhouse–Geisser correction and multiple comparison procedures. To statistically evaluate the research questions, the following null hypotheses were formulated at a 95% confidence level:

*H0₁*: There are no significant differences in item responses between teachers and pupils (Position).

*H0₂*: There are no significant differences in item responses with respect to the interaction between Position and Gender.

*H0₃*: There are no significant differences in item responses with respect to the interaction between Position and Experience (productive/receptive).

*H0₄*: There are no significant differences in item responses with respect to the interaction between Position and Study Programme.

### 3.3 Hypotheses testing

The hypotheses were tested using a repeated measures approach with the within-group factor ITEM and the between-group factor POSITION. This approach enabled the evaluation of differences in responses across questionnaire items while considering additional between-group factors such as Gender, Experience and Study Programme.

Profiles for individual items were subsequently created to analyse response patterns for different combinations of factors. Profiles for the combination ITEM*Position*Grade were not created due to the non-rejection of the corresponding global null hypothesis.

To verify the global null hypotheses, we applied adjusted tests for repeated measures (Greenhouse–Geisser adjustment, Table 1), due to the violation of the assumption of sphericity of the covariance matrix (Mauchly’s Sphericity Test: *p* < 0.001, Table 2). When the assumption of sphericity of the covariance matrix is not met, the Type I error rate increases.

**Table 1:** Greenhouse–Geisser adjustment test.

Effect	G-G Epsilon	G-G Adj. df1	G-G Adj. df2	G-G Adj. *p*
ITEM	0.7033	11.2523	5176.0650	0.0000
ITEM*Position	0.7033	11.2523	5176.0650	0.0000
ITEM*Position*Gender	0.7033	11.2523	5176.0650	0.0174
ITEM*Position*Experience (productive/receptive)	0.7046	22.5473	5163.3200	0.0484
ITEM*Position*Study Programme	0.6989	22.3645	5121.4620	0.0269
ITEM*Position*Grade	0.7038	33.7847	5135.2750	0.8672

**Table 2:** Mauchly’s test of sphericity.

Effect	W	Chi-Sqr.	df	*p*
ITEM*Position*Gender	0.052113	1342.6870	135	0.0000
ITEM*Position*Experience (productive/receptive)	0.052401	1334.2870	135	0.0000
ITEM*Position*Study Programme	0.050426	1351.6690	135	0.0000
ITEM*Position*Grade	0.052133	1330.7000	135	0.0000

Epsilon represents the degree of violation of the sphericity assumption. When epsilon equals one, it signifies the fulfilment of the assumption. Conversely, the smaller the value, the greater the violation of the sphericity assumption.

During the testing of the global null hypotheses, the Epsilon values were less than one. The null hypotheses stating that respondents’ answers are independent of the following item and combinations of factors are rejected with a 95% confidence level:

- ITEM*Position,
- ITEM*Position*Gender,
- ITEM*Position*Experience (productive/receptive),
- ITEM*Position*Study Programme

On the contrary, we do not reject the hypothesis stating that respondents’ answers are independent of the combination of factors ITEM*Position*Grade.

### 3.4 Multiple comparisons

Table 3 provides an overview of the overall comparison of responses across all questionnaire items and summarizes the relative differences in their evaluation. In terms of overall multiple comparisons, seven homogeneous groups (**** – *p* > 0.05) were identified among the evaluated items. A statistically significant difference was observed, for example, between the item “Training – Procedures – Alcohol Use” and the remaining questionnaire items. Similarly, differences were identified between the item “Satisfaction – Workspace” and several other items, including “Training – PPE specification,” “Knowledge of responsibilities,” “Agreement with the statement,” “Theoretical knowledge,” “Training – Interest,” “Training – Risk analysis (practical work),” “Knowledge assessment,” “Ability to identify hazards,” and “Examples of good practice (accidents).”

**Table 3:** Multiple comparisons of all items.

Total	Scale	1	2	3	4	5	6	7
Training – Procedures – Alcohol Use	2.34	****						
Training – Interest	3.34		****					
Training – Risk Analysis (practical work)	3.59			****				
Knowledge Assessment	3.80				****			
Ability to Identify Hazards	3.82				****			
Examples of Good Practice (accidents)	3.85				****			
Satisfaction – Work Environment	4.05					****		
Satisfaction – Working Conditions	4.11					****	****	
Focus on Practical Experience	4.11					****	****	
Initial Training – Equipment Operation and Tool Handling	4.12					****	****	
Knowledge of Rights	4.16					****	****	****
Familiarization – Procedure (Workplace Accident)	4.17					****	****	****
Training – Comprehensibility	4.17					****	****	****
Theoretical Knowledge	4.19					****	****	****
Training – PPE Specification	4.25						****	****
Knowledge of Responsibilities	4.27						****	****
Agreement with the Statement	4.31							****

The symbol “****” denotes homogeneous groups of items for which no statistically significant differences were identified (*p* > 0.05). Conversely, items that do not appear within the same column represent statistically significant differences between their mean responses (*p* < 0.05). This approach allows both the identification of groups of items with similar responses and the detection of statistically significant differences between distinct groups.

Detailed response profiles for combinations of factors (Position, Gender, Experience and Study Programme) are presented in the following tables and appendices in order to provide a more comprehensive interpretation of the results. The following tables illustrate how teachers and pupils evaluated individual items, highlighting which aspects of OSH training were assessed most negatively and most positively, as well as whether statistically significant differences were observed or whether the responses formed homogeneous groups.

In terms of multiple comparisons, six and seven homogeneous groups (**** – *p* > 0.05) were identified in the responses to individual items within the factor Position (Tables 4, 5).

**Table 4:** Profile for the combination of factors ITEM*Position – Teacher.

Position = Teacher	Scale	1	2	3	4	5	6
Training – Interest	3.26	****					
Ability to Identify Hazards	3.52	****	****				
Knowledge Assessment	3.80		****	****			
Training – Risk Analysis (practical work)	3.91			****	****		
Focus on Practical Experience	3.91			****	****		
Knowledge of Rights	3.98			****	****	****	
Training – PPE Specification	4.02			****	****	****	
Theoretical Knowledge	4.07			****	****	****	
Satisfaction – Work Environment	4.10			****	****	****	
Training – Comprehensibility	4.13			****	****	****	
Examples of Good Practice (accidents)	4.14			****	****	****	
Knowledge of Responsibilities	4.18				****	****	
Satisfaction – Working Conditions	4.19				****	****	
Training – Procedures – Alcohol Use	4.19				****	****	
Familiarization – Procedure (Workplace Accident)	4.27					****	
Initial Training – Equipment Operation and Tool Handling	4.29					****	****
Agreement with the Statement	4.63						****

**Table 5:** Profile for the combination of factors ITEM*Position – Pupil.

Position = Pupil	Scale	1	2	3	4	5	6	7
Training – Procedures – Alcohol Use	1.90	****						
Training – Interest	3.36		****					
Training – Risk Analysis (practical work)	3.52		****					
Examples of Good Practice (accidents)	3.78			****				
Knowledge Assessment	3.80			****				
Ability to Identify Hazards	3.90			****	****			
Satisfaction – Work Environment	4.04				****	****		
Initial Training – Equipment Operation and Tool Handling	4.08				****	****	****	
Satisfaction – Working Conditions	4.09					****	****	
Familiarization – Procedure (Workplace Accident)	4.15					****	****	****
Focus on Practical Experience	4.16					****	****	****
Training – Comprehensibility	4.18					****	****	****
Knowledge of Rights	4.20					****	****	****
Theoretical Knowledge	4.22					****	****	****
Agreement with the Statement	4.24						****	****
Knowledge of Responsibilities	4.29							****
Training – PPE Specification	4.31							****

A statistically significant difference was observed, for example, between the item “Training – Interest” and most of the other evaluated items, with the exception of “Ability to identify hazards.”

Within the factor Position – Pupils (Table 5), the item “Training – Procedures – Alcohol Use” was evaluated significantly differently compared with the remaining items. Differences were also observed between the item “Training – Risk analysis” and several other evaluated aspects of training, except for “Training – Interest.”

Additional examined factors such as Gender, Experience and Study Programme are presented in combination with the Item and Position in the tables in Appendices B, C, and D for improved clarity and readability.

Appendix B shows the combination of the factor ITEM*Position*Gender. Statistically significant differences can be observed in female teachers, for example, in the responses between the item “Ability to identify hazards” and all other items except for the item “Training - Interest.” In the case of the Pupil – Female factor, however, there were not as many statistically significant differences compared to the previous case, specifically there were no significant differences in the responses to up to six questions, that is, responses form almost homogeneous groups. Statistically significant differences were clearly evidenced in the responses to items related to trainings, procedures in case of alcohol use, and all other responses to the remaining questions. This is clearly visible in the visualizations presented through the above-mentioned graphs. In the responses of male pupils, significant differences are evident in the question concerning procedures in case of alcohol use, similarly to what was observed among female pupils. On the other hand, among teachers, more significant differences were found in the responses to questions related to the interest in trainings and the ability to identify hazards. In many responses, no statistically significant differences were found, as shown in Appendix B.

Appendix C presents multiple comparisons of the factor ITEM*Position*Experience, where, as mentioned above, we understand this experience as productive in the case of teachers, specifically their teaching experience, and receptive in the case of pupils, which is related to their age. For teachers, experience 1 corresponds to a teaching experience of 0–5 years, 2 represents experience of 6–10 years, and 3 indicates experience of more than 10 years. For pupils, 1 corresponds to the age range of 14–16 years, 2 represents the age range of 17–18 years, and 3 indicates the age range of 19–20 years. In all age groups of pupils, we can observe statistically significant differences in responses to items related to training procedures related to alcohol use, as well as other items. When looking at the teaching experience, the most significant differences in responses were identified among teachers with more than 10 years of experience, specifically in the item addressing the interest in trainings and the ability to identify hazards, along with all the other items except for these two previously mentioned. In terms of the overall multiple comparisons of the factor Experience, we identified only four homogeneous groups among teachers, indicating small differences in responses.

Within the combination of the factor ITEM*Position*Study Programme (Appendix D), we identified statistically significant differences in the replies from pupils across all fields of study in responses related to trainings focused on procedures related to alcohol use and other items. Whereas among pupils in the field of hotel services and other services, there was a statistically significant difference in responses related to the interest in trainings and other items as well, no further statistically significant differences were observed in the remaining items. Thus, pupils in these fields did not have a strong opinion or predominantly agreed with the questions, as only 3 homogeneous groups were identified.

From the perspective of Position – Teacher, the most statistically significant differences were identified within the hotel services and other services in responses to questions related to the interest in trainings and the ability to identify hazards, as well as other items. They also expressed the most critical views about these items. The responses in Appendix C, related to the study programmes, show that teachers responded only within four homogeneous groups, that is, they replied in a similar manner.

## 4 Discussion

### 4.1 Comparison of the statistically significant result (QR1)

The analysis identified statistically significant differences in responses based on respondent type (pupil/teacher), gender, professional experience, and field of study (QR1). The item most critically assessed was: “Is the procedure and consequences for alcohol use in your workplace part of the training?” Despite legal obligations requiring employers to implement and communicate a clear alcohol policy, this area was perceived as insufficiently addressed. Respondents—particularly pupils and males in technical and IT disciplines—consistently rated this aspect lowest. In contrast, more experienced teachers demonstrated a better understanding of procedures related to alcohol detection, a pattern not observed among pupils.

Findings align with Oesterle et al. (33), who reported that workplaces lacking formal alcohol policies or those not enforcing a total prohibition were approximately three times more likely to observe increased alcohol consumption and deterioration in work conditions among young workers. Moreover, young adults who perceived alcohol as tolerated or encouraged were nearly five times more likely to consume it in the workplace. Roche et al. (34) concluded that organizational safety culture significantly contributes to reducing workplace alcohol use. Accordingly, implementing preventive programmes within schools is recommended. Furthermore, institutional alcohol policies should be an integral component of mandatory employee training.

### 4.2 Knowledge of workplace hazards (QR2)

Comprehensive OSH training must systematically address workplace hazards and risk assessment strategies. However, evidence suggests that vocational education students often receive insufficient instruction in these domains [Kordošová & Urdzíková (35)]. Teachers -particularly female educators in hotel and service sectors—assigned greater importance to hazard identification. Conversely, a study by Andersson (36) reported student satisfaction where supervisors consistently reviewed risks prior to task execution. According to Tureková et al. (37), while 64% of students could correctly identify major workplace risks, 33% either could not or were uncertain. These findings indicate a need for more structured integration of risk management content into OSH training programmes.

### 4.3 Training quality (QR3)

Both pupils and teachers evaluated the engagement level of OSH training as relatively low, with average scores ranging from 3.5 to 3.8. Female teachers expressed significantly more critical views than their male counterparts. Notably, participants in hotel and business-related fields reported lower engagement. However, training related to the operation of tools and equipment was evaluated most positively across groups.

**Figure 4:**
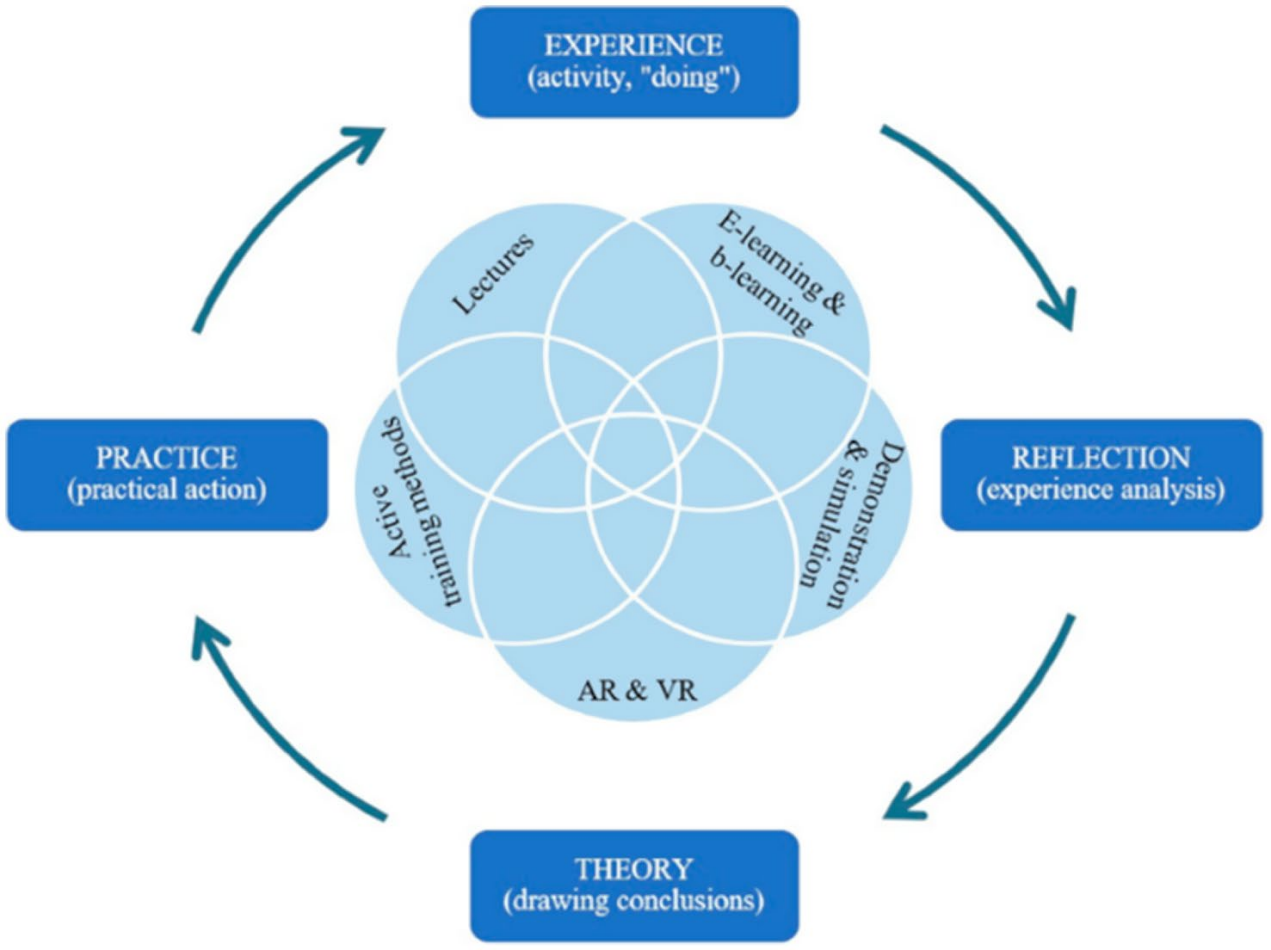
Training methods for safety in the Kolb learning cycle (45).

As shown by Boini et al. (38), participants who received OSH training during their education tended to report fewer work-related accidents. However, it should be emphasized that the present study primarily examines the perceptions of pupils and teachers regarding the quality and content of OSH training, rather than its direct impact on objective safety outcomes. The effectiveness of training is frequently associated in the literature with systematic planning, instructional design, and adequate preparation (39). In our study, respondents perceived the level of engagement during training as relatively low, which may indicate potential areas for improvement in the teaching methods used. High-quality OSH instruction should support the development of risk awareness and foster attitudes that contribute to long-term health protection (40, 41). Similarly, Barati Jozan et al. (42) suggest that increasing employees’ knowledge and competencies is associated with reductions in workplace accidents. Given the design of our research, these relationships are presented as theoretical context rather than as direct evidence of the effectiveness of the analysed training. Furthermore, studies highlight the superior impact of active training methodologies - including gamification, virtual reality, simulations, and role-playing—on learning outcomes (43, 44). Given the diverse and complex nature of OSH content, the application of mixed didactic strategies is recommended to ensure effective knowledge transfer (Figure 4) (45, 46).

Methods are among the most effective forms of teaching, enabling learners to achieve the knowledge and skills intended by the training objectives much more easily.

## 5 Conclusion

The evaluation involving 374 pupils and 90 teachers showed that although OSH training is conducted before entering the workplace, respondents often perceive it as insufficiently engaging and less effective in explaining risk assessment principles. It should be noted, however, that the results of this study reflect primarily the subjective perceptions and evaluations of the respondents regarding the quality of training, rather than objective measurements of safety outcomes or verified knowledge levels.

Pupils reported difficulties in identifying major workplace hazards and demonstrated limited familiarity with alcohol testing procedures, while teachers indicated a better understanding of these processes.

The study revealed differences in responses based on roles (pupils vs. teachers), study programmes, gender, grade level, and teaching experience. A key finding is the importance of OSH as a long-term educational topic. Teachers must understand basic OSH principles to influence pupils’ attitudes, especially during practical training. Interventions should target risks faced by young workers due to their developmental stage.

Integrating OSH training, management, and safety education into school curricula is crucial. Promoting a culture of prevention early increases the likelihood that students will recognize workplace hazards and adopt safer attitudes. Despite OSH being a cross-disciplinary theme in schools, most teachers do not encounter it during university studies and only gain awareness through workplace training. Many lack familiarity with OSH systems and risk assessment procedures, although those teaching practical subjects should introduce pupils to these areas.

Improving OSH education by adopting best practices from countries with established dual education systems may contribute to strengthening safety awareness among young workers. Policymakers, researchers, and practitioners must consider the complexities of training, work organization, and OSH culture. It should also be noted that the findings of this study are based on the specific context of the dual vocational education system in the Slovak Republic; therefore, their transferability to other countries may be limited due to differences in legislative, institutional, and organizational conditions related to vocational education and OSH systems. Training methods should be regularly evaluated by experts to enhance effectiveness. Continued efforts are needed to develop and implement effective ways of informing young employees about workplace risks and accident prevention.

These findings provide valuable guidance for decision-makers aiming to strengthen OSH education as a foundation for students’ future professional safety and health.

## Data Availability

The original contributions presented in the study are included in the article/Supplementary material, further inquiries can be directed to the corresponding author.
