# Correction: Investigating the level of education in the field of occupational safety and health in preparation for future profession: a Slovak case study

**DOI:** 10.3389/fpubh.2026.1863878

**Published:** 2026-05-06

**Authors:** Peter Brečka, Ivana Tureková, Michal Munk

**Affiliations:** 1 Department of Technology and Information Technologies, Faculty of Education, Constantine the Philosopher University, Nitra, Slovakia; 2 Department of Informatics, Faculty of Natural Sciences and Informatics, Constantine the Philosopher University, Nitra, Slovakia; 3 Science and Research Centre, University of Pardubice, Pardubice, Czechia

**Keywords:** pupils, questionnaire survey, risk management, teachers, training in occupational safety

In the published article, there was an error in the author affiliations.

The affiliations were incorrectly listed as:

Peter Brečka^1*^, Ivana Tureková^1^, Michal Munk^1,2^

^1^Department of Informatics, Faculty of Natural Sciences and Informatics, Constantine the Philosopher University, Nitra, Slovakia

^2^Science and Research Centre, University of Pardubice, Pardubice, Czechia

The correct affiliations are:

Peter Brečka^1*^, Ivana Tureková^1^ and Michal Munk^2,3^

^1^Department of Technology and Information Technologies, Faculty of Education, Constantine the Philosopher University, Nitra, Slovakia

^2^Department of Informatics, Faculty of Natural Sciences and Informatics, Constantine the Philosopher University, Nitra, Slovakia

^3^Science and Research Centre, University of Pardubice, Pardubice, Czechia

The original version of this article has been updated.

