# Supplementary material for: Investigating the level of education in the field of occupational safety and health in preparation for future profession: a Slovak case study
Source: Front Public Health. 2026 Apr 23;14:1744127. doi: 10.3389/fpubh.2026.1744127 (PMC13188965; doi:10.3389/fpubh.2026.1744127)
Supplement: Supplementary file 1 [file Supplementary_file_1.docx]

**Supplementary Material**

**Appendix A**

***Questionnaire for teachers and pupils used in the research section***

| **Teachers: Length of teaching experience (years)** | | **0-5** | **6-10** | | **More than 10 years** | | |
| --- | --- | --- | --- | --- | --- | --- | --- |
| **Pupils** | | **Study programme** | | | **Grade level** | | |
| **Gender** | | **male** | | | **female** | | |
| **No.** | **Survey question** | | **1*** | **2*** | **3*** | **4*** | **5*** |
|  | Do you think that pupils acquire sufficient theoretical knowledge in the field of occupational safety and health (OSH) during their studies? | |  |  |  |  |  |
|  | Is the level of theoretical OSH training sufficiently focused on practical application? | |  |  |  |  |  |
|  | Are pupils made adequately familiar with their rights regarding OSH? | |  |  |  |  |  |
|  | Are pupils made adequately familiar with their responsibilities regarding OSH? | |  |  |  |  |  |
|  | Are pupils familiarized with real-life examples of good practices to prevent accidents in their respective educational fields during their studies? | |  |  |  |  |  |
|  | Is familiarization with safe procedures for equipment operation and tool handling also part of the initial training? | |  |  |  |  |  |
|  | Do you consider the information from the trainings sufficiently understandable for pupils? | |  |  |  |  |  |
|  | Is the procedure and consequences of alcohol use at your workplace also included in the trainings? | |  |  |  |  |  |
|  | Is the result of risk analysis in practical work also part of the pupils' training? | |  |  |  |  |  |
|  | Do you think that pupils are able to identify the most significant hazards associated with the tasks performed in their respective professions? | |  |  |  |  |  |
|  | Do you consider the working conditions to be satisfactory in terms of occupational safety for pupils? | |  |  |  |  |  |
|  | Do you agree with the following statement: "Compliance with OSH regulations contributes to work efficiency, reduces the number of occupational accidents, and the occurrence of occupational diseases"? | |  |  |  |  |  |
|  | Is the precise specification of personal protective equipment resulting from risk analysis also part of the trainings? | |  |  |  |  |  |
|  | Do you consider the work environment to be suitable for the needs of pupils and performed work tasks? | |  |  |  |  |  |
|  | Do you think that OSH trainings are engaging and interesting for pupils? | |  |  |  |  |  |
|  | Is the procedure in case of a workplace accident also part of the training? | |  |  |  |  |  |
|  | Is the knowledge acquired by pupils from OSH trainings provably assessed (through tests, exams, etc.) prior to starting practical work? | |  |  |  |  |  |

**Appendix B**

***Profile for the combination of the factor ITEM*Position*Gender***

| **Position=Teacher. Gender=Women** | **Scale** | **1** | **2** | **3** | **4** | **5** |  |  | **Position=Pupil. Gender=Women** | **Scale** | **1** | **2** | **3** | **4** | **5** | **6** |
| --- | --- | --- | --- | --- | --- | --- | --- | --- | --- | --- | --- | --- | --- | --- | --- | --- |
| Training - Interest | 2.97 | **** |  |  |  |  |  |  | Training - Procedures - Alcohol Use | 2.22 | **** |  |  |  |  |  |
| Ability to Identify Hazards | 3.25 | **** | **** |  |  |  |  |  | Training - Interest | 3.42 |  | **** |  |  |  |  |
| Knowledge Assessment | 3.78 |  | **** | **** |  |  |  |  | Training - Risk Analysis (practical work) | 3.57 |  | **** | **** |  |  |  |
| Focus on Practical Experience | 3.81 |  | **** | **** |  |  |  |  | Examples of Good Practice (accidents) | 3.78 |  |  | **** | **** |  |  |
| Training - PPE Specification | 3.86 |  |  | **** | **** |  |  |  | Ability to Identify Hazards | 3.81 |  |  | **** | **** |  |  |
| Training - Risk Analysis (practical work) | 3.89 |  |  | **** | **** |  |  |  | Knowledge Assessment | 3.88 |  |  |  | **** | **** |  |
| Knowledge of Rights | 3.89 |  |  | **** | **** |  |  |  | Satisfaction - Work Environment | 4.02 |  |  |  | **** | **** | **** |
| Theoretical Knowledge | 3.92 |  |  | **** | **** |  |  |  | Satisfaction - Working Conditions | 4.05 |  |  |  | **** | **** | **** |
| Satisfaction - Work Environment | 3.97 |  |  | **** | **** |  |  |  | Initial Training - Equipment Operation and Tool Handling | 4.07 |  |  |  | **** | **** | **** |
| Training - Procedures - Alcohol Use | 4.00 |  |  | **** | **** |  |  |  | Focus on Practical Experience | 4.12 |  |  |  |  | **** | **** |
| Satisfaction - Working Conditions | 4.03 |  |  | **** | **** |  |  |  | Theoretical Knowledge | 4.17 |  |  |  |  | **** | **** |
| Training - Comprehensibility | 4.06 |  |  | **** | **** |  |  |  | Training - Comprehensibility | 4.18 |  |  |  |  | **** | **** |
| Familiarization - Procedure (Workplace Accident) | 4.06 |  |  | **** | **** |  |  |  | Knowledge of Rights | 4.19 |  |  |  |  |  | **** |
| Examples of Good Practice (accidents) | 4.08 |  |  | **** | **** |  |  |  | Familiarization - Procedure (Workplace Accident) | 4.24 |  |  |  |  |  | **** |
| Knowledge of Responsibilities | 4.19 |  |  | **** | **** | **** |  |  | Knowledge of Responsibilities | 4.26 |  |  |  |  |  | **** |
| Initial Training - Equipment Operation and Tool Handling | 4.42 |  |  |  | **** | **** |  |  | Agreement with the Statement | 4.28 |  |  |  |  |  | **** |
| Agreement with the Statement | 4.75 |  |  |  |  | **** |  |  | Training - PPE Specification | 4.31 |  |  |  |  |  | **** |
| **Position=Teacher. Gender=Man** | **Scale** | **1** | **2** | **3** | **4** | **5** | **6** |  | **Position=Pupil. Gender=Man** | **Scale** | **1** | **2** | **3** | **4** | **5** | **6** |
| Training - Interest | 3.44 | **** |  |  |  |  |  |  | Training - Procedures - Alcohol Use | 1.73 | **** |  |  |  |  |  |
| Ability to Identify Hazards | 3.70 | **** | **** |  |  |  |  |  | Training - Interest | 3.33 |  | **** |  |  |  |  |
| Knowledge Assessment | 3.81 | **** | **** | **** |  |  |  |  | Training - Risk Analysis (practical work) | 3.49 |  | **** |  |  |  |  |
| Training - Risk Analysis (practical work) | 3.93 |  | **** | **** | **** |  |  |  | Knowledge Assessment | 3.75 |  |  | **** |  |  |  |
| Focus on Practical Experience | 3.98 |  | **** | **** | **** |  |  |  | Examples of Good Practice (accidents) | 3.78 |  |  | **** |  |  |  |
| Knowledge of Rights | 4.04 |  | **** | **** | **** | **** |  |  | Ability to Identify Hazards | 3.94 |  |  | **** | **** |  |  |
| Training - PPE Specification | 4.13 |  |  | **** | **** | **** |  |  | Satisfaction - Work Environment | 4.05 |  |  |  | **** | **** |  |
| Knowledge of Responsibilities | 4.17 |  |  | **** | **** | **** | **** |  | Initial Training - Equipment Operation and Tool Handling | 4.08 |  |  |  | **** | **** | **** |
| Theoretical Knowledge | 4.17 |  |  | **** | **** | **** | **** |  | Familiarization - Procedure (Workplace Accident) | 4.10 |  |  |  | **** | **** | **** |
| Training - Comprehensibility | 4.19 |  |  | **** | **** | **** | **** |  | Satisfaction - Working Conditions | 4.11 |  |  |  | **** | **** | **** |
| Examples of Good Practice (accidents) | 4.19 |  |  | **** | **** | **** | **** |  | Focus on Practical Experience | 4.18 |  |  |  |  | **** | **** |
| Satisfaction - Work Environment | 4.19 |  |  | **** | **** | **** | **** |  | Training - Comprehensibility | 4.19 |  |  |  |  | **** | **** |
| Initial Training - Equipment Operation and Tool Handling | 4.20 |  |  | **** | **** | **** | **** |  | Knowledge of Rights | 4.20 |  |  |  |  | **** | **** |
| Satisfaction - Working Conditions | 4.30 |  |  |  | **** | **** | **** |  | Agreement with the Statement | 4.21 |  |  |  |  | **** | **** |
| Training - Procedures - Alcohol Use | 4.31 |  |  |  | **** | **** | **** |  | Theoretical Knowledge | 4.25 |  |  |  |  | **** | **** |
| Familiarization - Procedure (Workplace Accident) | 4.41 |  |  |  |  | **** | **** |  | Knowledge of Responsibilities | 4.31 |  |  |  |  |  | **** |
| Agreement with the Statement | 4.56 |  |  |  |  |  | **** |  | Training - PPE Specification | 4.31 |  |  |  |  |  | **** |

**Appendix C**

***Profile for the combination of factor ITEM*Position*Experience***

| **Position=Teacher. Experience** | **Scale** | **1** | **2** | **3** | **4** |  | **Position=Pupil. Experience** | **Scale** | **1** | **2** | **3** | **4** | **5** |  |
| --- | --- | --- | --- | --- | --- | --- | --- | --- | --- | --- | --- | --- | --- | --- |
| **(productive/receptive)=1.** |  |  |  |  |  |  | **(productive/receptive)=1.** |  |  |  |  |  |  |  |
| Training - Interest | 3.26 | **** |  |  |  |  | Training - Procedures - Alcohol Use | 1.71 | **** |  |  |  |  |  |
| Ability to Identify Hazards | 3.52 | **** | **** |  |  |  | Training - Interest | 3.57 |  | **** |  |  |  |  |
| Knowledge of Rights | 3.78 | **** | **** | **** |  |  | Training - Risk Analysis (practical work) | 3.61 |  | **** | **** |  |  |  |
| Focus on Practical Experience | 3.83 | **** | **** | **** |  |  | Knowledge Assessment | 3.84 |  | **** | **** |  |  |  |
| Knowledge Assessment | 3.83 | **** | **** | **** |  |  | Ability to Identify Hazards | 3.87 |  |  | **** |  |  |  |
| Theoretical Knowledge | 4.00 |  | **** | **** | **** |  | Examples of Good Practice (accidents) | 3.89 |  |  | **** | **** |  |  |
| Examples of Good Practice (accidents) | 4.04 |  | **** | **** | **** |  | Initial Training - Equipment Operation and Tool Handling | 4.15 |  |  |  | **** | **** |  |
| Training - Risk Analysis (practical work) | 4.09 |  | **** | **** | **** |  | Satisfaction - Working Conditions | 4.21 |  |  |  |  | **** |  |
| Training - Procedures - Alcohol Use | 4.17 |  | **** | **** | **** |  | Familiarization - Procedure (Workplace Accident) | 4.21 |  |  |  |  | **** |  |
| Training - Comprehensibility | 4.26 |  |  | **** | **** |  | Satisfaction - Work Environment | 4.23 |  |  |  |  | **** |  |
| Knowledge of Responsibilities | 4.30 |  |  | **** | **** |  | Focus on Practical Experience | 4.25 |  |  |  |  | **** |  |
| Satisfaction - Work Environment | 4.30 |  |  | **** | **** |  | Training - Comprehensibility | 4.29 |  |  |  |  | **** |  |
| Training - PPE Specification | 4.35 |  |  | **** | **** |  | Knowledge of Rights | 4.30 |  |  |  |  | **** |  |
| Satisfaction - Working Conditions | 4.35 |  |  | **** | **** |  | Knowledge of Responsibilities | 4.36 |  |  |  |  | **** |  |
| Familiarization - Procedure (Workplace Accident) | 4.39 |  |  | **** | **** |  | Agreement with the Statement | 4.38 |  |  |  |  | **** |  |
| Initial Training - Equipment Operation and Tool Handling | 4.43 |  |  | **** | **** |  | Theoretical Knowledge | 4.40 |  |  |  |  | **** |  |
| Agreement with the Statement | 4.61 |  |  |  | **** |  | Training - PPE Specification | 4.40 |  |  |  |  | **** |  |
| **Position=Teacher. Experience** | **Scale** | **1** | **2** | **3** | **4** |  | **Position=Pupil. Experience** | **Scale** | **1** | **2** | **3** | **4** | **5** | **6** |
| **(productive/receptive)=2.** |  |  |  |  |  |  | **(productive/receptive)=2.** |  |  |  |  |  |  |  |
| Training - Interest | 3.00 | **** |  |  |  |  | Training - Procedures - Alcohol Use | 1.93 | **** |  |  |  |  |  |
| Ability to Identify Hazards | 3.27 | **** | **** |  |  |  | Training - Interest | 3.23 |  | **** |  |  |  |  |
| Knowledge Assessment | 3.33 | **** | **** | **** |  |  | Training - Risk Analysis (practical work) | 3.49 |  | **** |  |  |  |  |
| Training - Risk Analysis (practical work) | 3.60 | **** | **** | **** | **** |  | Examples of Good Practice (accidents) | 3.75 |  |  | **** |  |  |  |
| Training - PPE Specification | 3.67 | **** | **** | **** | **** |  | Knowledge Assessment | 3.86 |  |  | **** | **** |  |  |
| Satisfaction - Work Environment | 3.80 | **** | **** | **** | **** |  | Ability to Identify Hazards | 3.93 |  |  | **** | **** | **** |  |
| Theoretical Knowledge | 3.93 |  | **** | **** | **** |  | Satisfaction - Work Environment | 3.94 |  |  | **** | **** | **** |  |
| Training - Comprehensibility | 4.07 |  | **** | **** | **** |  | Satisfaction - Working Conditions | 4.04 |  |  |  | **** | **** | **** |
| Focus on Practical Experience | 4.07 |  | **** | **** | **** |  | Initial Training - Equipment Operation and Tool Handling | 4.05 |  |  |  | **** | **** | **** |
| Knowledge of Rights | 4.07 |  | **** | **** | **** |  | Focus on Practical Experience | 4.14 |  |  |  |  | **** | **** |
| Satisfaction - Working Conditions | 4.07 |  | **** | **** | **** |  | Familiarization - Procedure (Workplace Accident) | 4.14 |  |  |  |  | **** | **** |
| Knowledge of Responsibilities | 4.13 |  | **** | **** | **** |  | Theoretical Knowledge | 4.14 |  |  |  |  | **** | **** |
| Initial Training - Equipment Operation and Tool Handling | 4.20 |  |  | **** | **** |  | Agreement with the Statement | 4.16 |  |  |  |  | **** | **** |
| Examples of Good Practice (accidents) | 4.20 |  |  | **** | **** |  | Training - Comprehensibility | 4.16 |  |  |  |  | **** | **** |
| Familiarization - Procedure (Workplace Accident) | 4.27 |  |  |  | **** |  | Knowledge of Rights | 4.18 |  |  |  |  | **** | **** |
| Training - Procedures - Alcohol Use | 4.33 |  |  |  | **** |  | Training - PPE Specification | 4.28 |  |  |  |  |  | **** |
| Agreement with the Statement | 4.47 |  |  |  | **** |  | Knowledge of Responsibilities | 4.30 |  |  |  |  |  | **** |
| **Position=Teacher. Experience** | **Scale** | **1** | **2** | **3** | **4** |  | **Position=Pupil. Experience** | **Scale** | **1** | **2** | **3** | **4** | **5** | **6** |
| **(productive/receptive)=3.** |  |  |  |  |  |  | **(productive/receptive)=3.** |  |  |  |  |  |  |  |
| Training - Interest | 3.33 | **** |  |  |  |  | Training - Procedures - Alcohol Use | 2.27 | **** |  |  |  |  |  |
| Ability to Identify Hazards | 3.60 | **** | **** |  |  |  | Training - Interest | 3.23 |  | **** |  |  |  |  |
| Focus on Practical Experience | 3.90 |  | **** | **** |  |  | Training - Risk Analysis (practical work) | 3.37 |  | **** | **** |  |  |  |
| Training - Risk Analysis (practical work) | 3.92 |  | **** | **** |  |  | Knowledge Assessment | 3.46 |  | **** | **** | **** |  |  |
| Knowledge Assessment | 3.92 |  | **** | **** |  |  | Examples of Good Practice (accidents) | 3.60 |  | **** | **** | **** | **** |  |
| Training - PPE Specification | 3.98 |  | **** | **** |  |  | Satisfaction - Work Environment | 3.83 |  |  | **** | **** | **** | **** |
| Knowledge of Rights | 4.04 |  |  | **** |  |  | Ability to Identify Hazards | 3.85 |  |  | **** | **** | **** | **** |
| Satisfaction - Work Environment | 4.10 |  |  | **** |  |  | Satisfaction - Working Conditions | 3.90 |  |  | **** | **** | **** | **** |
| Training - Comprehensibility | 4.10 |  |  | **** |  |  | Focus on Practical Experience | 3.94 |  |  |  | **** | **** | **** |
| Knowledge of Responsibilities | 4.13 |  |  | **** |  |  | Training - Comprehensibility | 3.96 |  |  |  | **** | **** | **** |
| Theoretical Knowledge | 4.13 |  |  | **** |  |  | Initial Training - Equipment Operation and Tool Handling | 3.96 |  |  |  | **** | **** | **** |
| Training - Procedures - Alcohol Use | 4.15 |  |  | **** |  |  | Knowledge of Rights | 3.98 |  |  |  | **** | **** | **** |
| Satisfaction - Working Conditions | 4.15 |  |  | **** |  |  | Familiarization - Procedure (Workplace Accident) | 4.00 |  |  |  | **** | **** | **** |
| Examples of Good Practice (accidents) | 4.17 |  |  | **** |  |  | Theoretical Knowledge | 4.00 |  |  |  | **** | **** | **** |
| Familiarization - Procedure (Workplace Accident) | 4.21 |  |  | **** |  |  | Knowledge of Responsibilities | 4.08 |  |  |  |  | **** | **** |
| Initial Training - Equipment Operation and Tool Handling | 4.25 |  |  | **** |  |  | Agreement with the Statement | 4.12 |  |  |  |  | **** | **** |
| Agreement with the Statement | 4.69 |  |  |  | **** |  | Training - PPE Specification | 4.17 |  |  |  |  |  | **** |

**Appendix D**

***Profile for the combination of factor ITEM*Position*Study Programme***

| **Position=Teacher** | **Scale** | **1** | **2** | **3** | **4** |  | **Position=Pupil** | **Scale** | **1** | **2** | **3** | **4** | **5** | **6** |  |
| --- | --- | --- | --- | --- | --- | --- | --- | --- | --- | --- | --- | --- | --- | --- | --- |
| **Study Programme=Technical and Information Technology Services** |  |  |  |  |  |  | **Study Programme=Technical and Information Technology Services** |  |  |  |  |  |  |  |  |
| Training - Interest | 3.58 | **** |  |  |  |  | Training - Procedures - Alcohol Use | 1,75 | **** |  |  |  |  |  |  |
| Ability to Identify Hazards | 3.69 | **** | **** |  |  |  | Training - Interest | 3.29 |  | **** |  |  |  |  |  |
| Focus on Practical Experience | 3.98 | **** | **** | **** |  |  | Training - Risk Analysis (practical work) | 3.49 |  | **** |  |  |  |  |  |
| Knowledge Assessment | 3.98 | **** | **** | **** |  |  | Knowledge Assessment | 3.77 |  |  | **** |  |  |  |  |
| Training - Risk Analysis (practical work) | 4.02 |  | **** | **** |  |  | Examples of Good Practice (accidents) | 3.78 |  |  | **** |  |  |  |  |
| Satisfaction - Work Environment | 4.08 |  | **** | **** |  |  | Ability to Identify Hazards | 3.90 |  |  | **** | **** |  |  |  |
| Theoretical Knowledge | 4.12 |  |  | **** |  |  | Satisfaction - Work Environment | 4.03 |  |  |  | **** | **** |  |  |
| Knowledge of Rights | 4.13 |  |  | **** |  |  | Initial Training - Equipment Operation and Tool Handling | 4.05 |  |  |  | **** | **** | **** |  |
| Satisfaction - Working Conditions | 4.15 |  |  | **** |  |  | Satisfaction - Working Conditions | 4.07 |  |  |  | **** | **** | **** |  |
| Training - PPE Specification | 4.15 |  |  | **** |  |  | Familiarization - Procedure (Workplace Accident) | 4.09 |  |  |  | **** | **** | **** |  |
| Examples of Good Practice (accidents) | 4.19 |  |  | **** | **** |  | Focus on Practical Experience | 4.13 |  |  |  |  | **** | **** |  |
| Training - Comprehensibility | 4.19 |  |  | **** | **** |  | Training - Comprehensibility | 4.16 |  |  |  |  | **** | **** |  |
| Initial Training - Equipment Operation and Tool Handling | 4.23 |  |  | **** | **** |  | Knowledge of Rights | 4.16 |  |  |  |  | **** | **** |  |
| Knowledge of Responsibilities | 4.27 |  |  | **** | **** |  | Agreement with the Statement | 4.19 |  |  |  |  | **** | **** |  |
| Training - Procedures - Alcohol Use | 4.33 |  |  | **** | **** |  | Theoretical Knowledge | 4.20 |  |  |  |  | **** | **** |  |
| Familiarization - Procedure (Workplace Accident) | 4.38 |  |  | **** | **** |  | Training - PPE Specification | 4.25 |  |  |  |  | **** | **** |  |
| Agreement with the Statement | 4.58 |  |  |  | **** |  | Knowledge of Responsibilities | 4.28 |  |  |  |  |  | **** |  |
| **Position=Teacher** | **Scale** | **1** | **2** | **3** | **4** |  | **Position=Pupil** | **Scale** | **1** | **2** | **3** | **4** | **5** | **6** | **7** |
| **Study Programme=Business Services** |  |  |  |  |  |  | **Study Programme=Business Services** |  |  |  |  |  |  |  |  |
| Training - Interest | 2.94 | **** |  |  |  |  | Training - Procedures - Alcohol Use | 2.15 | **** |  |  |  |  |  |  |
| Ability to Identify Hazards | 3.33 | **** | **** |  |  |  | Training - Risk Analysis (practical work) | 3.50 |  | **** |  |  |  |  |  |
| Knowledge Assessment | 3.50 | **** | **** | **** |  |  | Training - Interest | 3.63 |  | **** | **** |  |  |  |  |
| Focus on Practical Experience | 3.72 | **** | **** | **** |  |  | Examples of Good Practice (accidents) | 3.79 |  | **** | **** | **** |  |  |  |
| Training - Risk Analysis (practical work) | 3.83 | **** | **** | **** | **** |  | Knowledge Assessment | 3.84 |  | **** | **** | **** | **** |  |  |
| Training - Procedures - Alcohol Use | 3.83 | **** | **** | **** | **** |  | Ability to Identify Hazards | 3.86 |  |  | **** | **** | **** | **** |  |
| Training - PPE Specification | 3.89 |  | **** | **** | **** |  | Satisfaction - Working Conditions | 4.11 |  |  |  | **** | **** | **** | **** |
| Knowledge of Rights | 3.89 |  | **** | **** | **** |  | Satisfaction - Work Environment | 4.12 |  |  |  | **** | **** | **** | **** |
| Knowledge of Responsibilities | 3.89 |  | **** | **** | **** |  | Initial Training - Equipment Operation and Tool Handling | 4.17 |  |  |  |  | **** | **** | **** |
| Familiarization - Procedure (Workplace Accident) | 3.89 |  | **** | **** | **** |  | Focus on Practical Experience | 4.21 |  |  |  |  |  | **** | **** |
| Theoretical Knowledge | 3.94 |  | **** | **** | **** |  | Training - Comprehensibility | 4.25 |  |  |  |  |  |  | **** |
| Training - Comprehensibility | 3.94 |  | **** | **** | **** |  | Theoretical Knowledge | 4.28 |  |  |  |  |  |  | **** |
| Examples of Good Practice (accidents) | 4.06 |  | **** | **** | **** |  | Knowledge of Rights | 4.29 |  |  |  |  |  |  | **** |
| Satisfaction - Work Environment | 4.17 |  | **** | **** | **** |  | Knowledge of Responsibilities | 4.32 |  |  |  |  |  |  | **** |
| Satisfaction - Working Conditions | 4.22 |  | **** | **** | **** |  | Familiarization - Procedure (Workplace Accident) | 4.32 |  |  |  |  |  |  | **** |
| Initial Training - Equipment Operation and Tool Handling | 4.39 |  |  | **** | **** |  | Agreement with the Statement | 4.32 |  |  |  |  |  |  | **** |
| Agreement with the Statement | 4.67 |  |  |  | **** |  | Training - PPE Specification | 4.45 |  |  |  |  |  |  | **** |
| **Position=Teacher** | **Scale** | **1** | **2** | **3** | **4** |  | **Position=Pupil** | **Scale** | **1** | **2** | **3** |  |  |  |  |
| **Study Programme=Hotel Services and Other Services** |  |  |  |  |  |  | **Study Programme=Hotel Services and Other Services** |  |  |  |  |  |  |  |  |
| Training - Interest | 2.70 | **** |  |  |  |  | Training - Procedures - Alcohol Use | 2.25 | **** |  |  |  |  |  |  |
| Ability to Identify Hazards | 3.25 | **** | **** |  |  |  | Training - Interest | 3.07 |  | **** |  |  |  |  |  |
| Knowledge Assessment | 3.60 |  | **** | **** |  |  | Training - Risk Analysis (practical work) | 3.75 |  |  | **** |  |  |  |  |
| Knowledge of Rights | 3.65 |  | **** | **** |  |  | Examples of Good Practice (accidents) | 3.75 |  |  | **** |  |  |  |  |
| Training - Risk Analysis (practical work) | 3.70 |  | **** | **** |  |  | Satisfaction - Work Environment | 3.82 |  |  | **** |  |  |  |  |
| Training - PPE Specification | 3.80 |  | **** | **** |  |  | Knowledge Assessment | 3.86 |  |  | **** |  |  |  |  |
| Focus on Practical Experience | 3.90 |  | **** | **** |  |  | Initial Training - Equipment Operation and Tool Handling | 3.96 |  |  | **** |  |  |  |  |
| Theoretical Knowledge | 4.05 |  | **** | **** | **** |  | Familiarization - Procedure (Workplace Accident) | 4.00 |  |  | **** |  |  |  |  |
| Examples of Good Practice (accidents) | 4.10 |  |  | **** | **** |  | Ability to Identify Hazards | 4.00 |  |  | **** |  |  |  |  |
| Satisfaction - Work Environment | 4.10 |  |  | **** | **** |  | Focus on Practical Experience | 4.14 |  |  | **** |  |  |  |  |
| Training - Comprehensibility | 4.15 |  |  | **** | **** |  | Satisfaction - Working Conditions | 4.14 |  |  | **** |  |  |  |  |
| Training - Procedures - Alcohol Use | 4.15 |  |  | **** | **** |  | Theoretical Knowledge | 4.18 |  |  | **** |  |  |  |  |
| Knowledge of Responsibilities | 4.20 |  |  | **** | **** |  | Training - Comprehensibility | 4.21 |  |  | **** |  |  |  |  |
| Satisfaction - Working Conditions | 4.25 |  |  | **** | **** |  | Knowledge of Rights | 4.25 |  |  | **** |  |  |  |  |
| Familiarization - Procedure (Workplace Accident) | 4.30 |  |  | **** | **** |  | Knowledge of Responsibilities | 4.32 |  |  | **** |  |  |  |  |
| Initial Training - Equipment Operation and Tool Handling | 4.35 |  |  | **** | **** |  | Training - PPE Specification | 4.32 |  |  | **** |  |  |  |  |
| Agreement with the Statement | 4.75 |  |  |  | **** |  | Agreement with the Statement | 4.36 |  |  | **** |  |  |  |  |
